# Rupture of the extensor pollicis longus tendon following dorsal entry flexible nailing of radial shaft fractures in children

**DOI:** 10.1007/s11832-014-0605-0

**Published:** 2014-08-07

**Authors:** Ben Brooker, P. Christian Harris, Leo T. Donnan, H. Kerr Graham

**Affiliations:** 1 Orthopaedic Department, The Royal Children’s Hospital, Flemington Road, Parkville, Melbourne, VIC 3052 Australia; 2 Orthopaedic Department, Southern Health, Dandenong Hospital, Dandenong, VIC Australia; 3 Orthopaedic Department, Sunshine Hospital, St. Albans, VIC Australia; 4 The University of Melbourne, Parkville, VIC Australia; 5 Murdoch Childrens Research Institute, Parkville, VIC Australia

**Keywords:** Radial fracture, Flexible nailing, Tendon rupture

## Abstract

**Introduction:**

Diaphyseal forearm fractures are common in children and adolescents. Intramedullary fixation with flexible nails has a high success rate. Complications related to the insertion of the radial nail include injury to the superficial branch of the radial nerve and rupture of the extensor pollicis longus (EPL) tendon.

**Materials and Methods:**

We report a series of nine patients who sustained an EPL injury related to the insertion of an elastic intramedullary nail into the radius.

**Results:**

All nine patients underwent operative management, consisting of either EPL release, EPL direct repair, or tendon transfer (using extensor indicis proprius). In all cases, the nail entry site was directly related to the location of EPL. In many of the cases the EPL dysfunction occurred early on but it’s recognition was often delayed.

**Conclusion:**

Based on our findings, we recommend the use of a radial entry point. For surgeons who prefer the dorsal entry point, we recommend that they use an incision which allows visualisation of the extensor tendons and that any post-operative EPL dysfunction is addressed promptly.

## Introduction

Fractures of the radius and ulna are common in children. Whilst closed reduction and immobilisation in an above-elbow cast is the most widely used treatment, not all fractures can be reduced adequately by closed manipulation and not all reductions can be maintained by a cast. Internal fixation with elastic stable intramedullary nailing (ESIN) has found increasing popularity [1, 2]. Not only can it improve the quality of a closed reduction but it also increases the stability of the fracture, resulting in a shorter time in cast and a lower risk of malunion. Disadvantages of ESIN relate to the invasive nature and include the potential for injury to structures at the nail entry points, infection (superficial and deep), plus the need for subsequent surgery for implant removal. In the majority of studies, the incidence of surgical complications has been low and most are mild and self-limiting [1–3]. The most common complications include irritation from the exposed ends of the nails and difficulties with implant removal [4, 5].

The radial nail is inserted retrograde, via the distal metaphysis. Injury to the distal radial physis is avoided by choosing an entry point proximal to the physis using fluoroscopy.

Suggested entry points include dorsal or radial. With the radial approach, the superficial branches of the radial nerve are at risk. With the dorsal approach, the extensor tendons are at risk, especially the extensor pollicis longus (EPL). For this reason, the interval between the first and second extensor compartments has been recommended [1, 2].

The EPL tendon has a tenuous blood supply. Delayed rupture has been reported after both displaced and undisplaced fractures of the distal radius, in adults and in children [6–8]. Delayed rupture has also been reported after closed treatment and after internal fixation with various devices, including volar locking plates [9, 10]. However, rupture of the EPL tendon in children is rare and, as a complication of flexible nail insertion, has been only occasionally reported [11]. To our knowledge, there are only nine cases in the English literature, five of which were not reported in detail [2, 11, 12]. We report our experience of this complication, make recommendations regarding the choice of nail entry point and highlight the importance of early recognition of EPL dysfunction after radial ESIN.

## Patients and methods

**Fig. 1:**
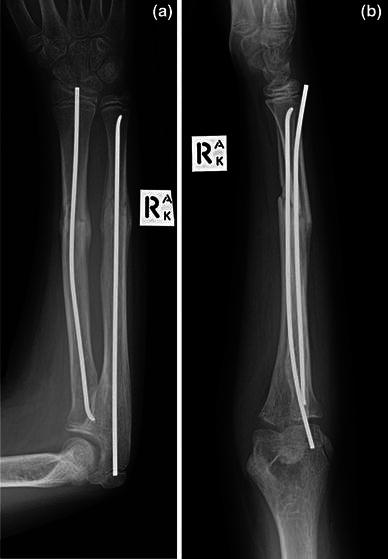
Anteroposterior and lateral radiographs representing the typical nail entry sites for all cases

Over a 5-year period (December 2008 to July 2013), nine cases with delayed rupture or dysfunction of the EPL following intramedullary nailing of forearm fractures in paediatric patients were identified. Patients were referred from a number of hospitals in the metropolitan area to a paediatric tertiary care centre. All patients had displaced diaphyseal fractures of the radius and ulna. In each case, the initial surgeon had inserted the radial nail in a percutaneous manner; they had identified the entry level using fluoroscopy, made a small dorsal skin incision (approximately 5 mm) over Lister’s tubercle, performed blunt dissection down to bone and used an awl to make the entry point, without visualisation of the extensor tendons. Standard approaches had been used, both for open fractures and for those requiring open reduction. The nails had been cut short, just below skin level, to permit later removal (Fig. 1). The incisions were closed with a single absorbable suture and the upper limb immobilised in an above-elbow plaster cast. Patients were admitted to hospital for elevation and neurovascular observation, and were typically discharged the following day.

## Results

There were six males and three females, with a median age of 12 years (range 9–14). In all cases, both forearm bones were involved. Flexible nailing was the primary procedure in seven patients, performed within 36 h of the injury. In the other two cases, flexible nailing was performed for loss of position after closed reduction and casting. Six of the nine operations were performed by consultant surgeons and three cases were performed by registrars, without consultant supervision. Six of the cases were performed during “in hours” operating lists, with three cases performed after hours (Table 1).

**Table 1:** Demographics and fracture management

Case	Age (years)	Gender	Side	Fracture details	Injury to surgery (days)	ESIN	Surgeon	Surgery time
1	13	M	Left	Closed	0	Radius (OR), ulna (CR)	Consultant	In hours
2	9	M	Right	Closed	1	Radius (OR)	Consultant	Evening
3	10	F	Right	Open (radius)	1	Radius (OR), ulna (CR)	Consultant	In hours
4	13	M	Left	Open (ulna)	0	Radius (CR), ulna (OR)	Consultant	Evening
5	11	F	Left	Closed	12	Radius (CR), ulna (CR)	Consultant	In hours
6	12	M	Left	Closed	1	Radius (OR)	Registrar	In hours
7	13	M	Right	Closed	7	Radius (CR), ulna (CR)	Consultant	In hours
8	12	F	Left	Closed	1	Radius (CR), ulna (CR)	Registrar	In hours
9	14	M	Left	Closed	0	Radius (CR), ulna (CR)	Registrar	Evening

*OR* open reduction, *CR* closed reduction

Eight patients had an uncomplicated post-operative course and were discharged the day after surgery. One patient was noted to have pain, swelling and inability to extend his thumb, a finding that was misdiagnosed as a posterior interosseous nerve (PIN) palsy, and his discharge was delayed. All patients received regular outpatient review. Dysfunction of thumb extension was noticed early in six patients but the significance was recognised in only one patient who underwent early surgery. The median time from nail insertion to the diagnosis of EPL injury was 80 days (range 7–159). All diagnoses were made on clinical grounds, although ultrasound examination was performed in five patients in an attempt to confirm the diagnosis prior to surgery (Table 2).

**Table 2:** EPL dysfunction and management

Case	Early features of EPL dysfunction	Index operation to diagnosis of EPL dysfunction (days)	Ultrasound examination performed	Index operation to EPL surgery (days)	Type of treatment
1	No	56	No	74	EI-EPL transfer
2	Yes	159	Yes	170	EPL repair
3	Yes	84	Yes	105	EI-EPL transfer
4	Yes	43	No	62	EPL release
5	Yes	80	Yes	85	EPL repair
6	No	53	No	55	EI-EPL transfer
7	Yes	7	Yes	7	EPL release
8	Yes	144	No	166	EI-EPL transfer
9	No	85	Yes	152	EPL release

*EI* extensor indicis, *EPL* extensor pollicis longus

**Fig. 2:**
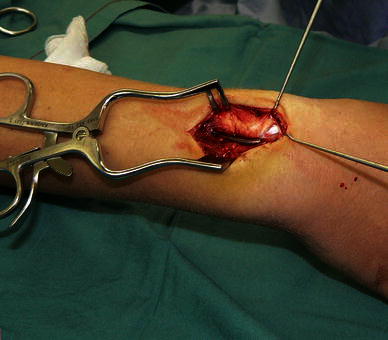
Case 7: EPL tendon compressed under nail

**Fig. 3:**
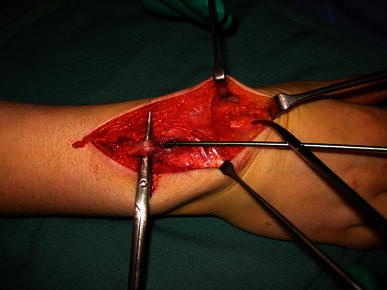
Case 2: relationship between EPL rupture and nail entry site

All patients underwent operative management. In three patients, the EPL tendon was intact but trapped (compressed) by the end of the nail, where it exited from the bone. Decompression was performed in one patient by bending the nail end away from the tendon, allowing it to glide freely (Fig. 2). In two other patients, the nail was removed and the EPL tendon released. EPL function rapidly returned to normal in these three patients. In the other six patients. there was complete rupture of the EPL tendon, which was treated by extensor indicis proprius transfer in four patients and direct EPL repair in two patients. Nails were removed at the same time (Fig. 3). The reconstruction or repair was protected by casting with the thumb extended for 4–6 weeks, followed by rehabilitation exercises, supervised by a hand therapist. The functional outcomes were good at a mean follow up of 14 months.

## Discussion

In the early 1980s, a group from France first described ESIN for diaphyseal fractures of the forearm bones [1, 13]. Their entry point for the radius was direct lateral (radial). Whilst this risks injury to the superficial radial nerve [2, 4, 5, 14–17], most reports of this complication state the return of sensation to normal within a few months. Although there is a risk of a symptomatic neuroma, none have been reported in association with ESIN [18]. Since then, some authors have recommended a dorsal entry point for particular fractures, believing that the forces produced by the nail in this position gives better control of the fracture [19, 20]. This, however, risks injury to the extensor tendons and, in particular, the EPL.

The EPL tendon has a tenuous blood supply and is prone to delayed rupture after fractures of the distal radius in adults [6]. The mechanism of delayed rupture cannot solely be attrition across the sharp bone edge because rupture has been reported after undisplaced fractures of the distal radius, in both adults and in children [7, 8]. Fracture displacement and internal fixation increase the risk of delayed rupture. Injury from drills and attrition from prominent screw tips may be contributory factors [9, 10].

In children, the risk factors are quite different. Delayed rupture of the EPL is rare after distal radial fractures but seems to be increasing following ESIN for diaphyseal fractures of the radius [3]. ESIN is generally considered a minimally invasive procedure [21], with low rates of complications [1, 2, 4, 14]. A recent large study of complications and outcomes of diaphyseal forearm fractures reported good to excellent outcomes in 91 % of fractures, with a 17 % rate of grade 2–4 complications according to a new classification system [22]. In this series of 205 fractures treated by ESIN, the insertion of the radial nail was radial in 61 % and dorsal in 39 %. The authors reported four superficial radial nerve injuries, all of which resolved with observation. However, they reported only one EPL rupture, associated with the removal of a radial nail from the dorsal approach. The authors reported this as a grade 4 complication because it had the potential for resulting in a permanent deficit. Arguably, EPL ruptures could be reported as a grade 3 complication, i.e. requiring inpatient management or re-operation.

A review of the literature identified nine previously reported cases of EPL rupture in association with dorsal entry site ESIN in children [2, 4, 11, 12, 23–25], although the majority of these were not reported in detail [2, 11, 12]. In our series, neither surgeon experience nor fatigue were contributory factors but, rather, it is the “blind” dorsal entry site that is inherently risky. The EPL tendon may be trapped, impaled or undergo delayed rupture from attrition or ischaemia.

Given that a number of mechanisms for rupture have been postulated and observed in this series, there is clearly a role for increased awareness, earlier recognition and earlier intervention. In three of our patients, the tendon was found at surgical exploration to be intact but entrapped under the protruding nail, with normal return of function after removal of the nail. In a further three cases, decreased thumb extension was an early clinical sign, suggesting that tethering of the tendon at the time of operation may have occurred with later rupture, and in one case, rupture was preceded by pain on thumb extension. Early clinical evaluation and a high index of suspicion may have allowed early diagnosis of these cases, possibly before tendon rupture occurred, and, thus, avoided reconstructive surgery and its associated rehabilitation.

Ultrasound examination was used in half of our patients but the results were sometimes misleading. If there is EPL dysfunction after dorsal insertion of a flexible nail, exploration, not ultrasound, is required.

The best way to avoid EPL injury during ESIN of the radius is to use a radial insertion point. If the surgeon prefers to use the dorsal approach, a larger incision may be required, as well as vigilance to detect early EPL dysfunction.

## Conclusion

Extensor pollicis longus (EPL) rupture following the insertion of an intramedullary nail for the management of a diaphyseal fracture of the radius may be more common than reported in the literature. To prevent this significant complication, we recommend a radial entry point.
